# Challenges for malaria elimination in Zanzibar: pyrethroid resistance in malaria vectors and poor performance of long-lasting insecticide nets

**DOI:** 10.1186/1756-3305-6-82

**Published:** 2013-03-28

**Authors:** Khamis A Haji, Bakari O Khatib, Stephen Smith, Abdullah S Ali, Gregor J Devine, Maureen Coetzee, Silas Majambere

**Affiliations:** 1 Zanzibar Malaria Control Program, Mwanakwerekwe, P.O. Box 407, Zanzibar, Tanzania; 2 Malaria Entomology Research Unit, School of Pathology, Faculty of Health Sciences, University of the Witwatersrand, 7 York Road, Parktown, Johannesburg, 2193, South Africa; 3 Centers for Disease Control and Prevention, 4770 Buford Highway, Atlanta, GA, 30341, USA; 4 Ifakara Health Institute, Dar es Salaam, P.O. Box 78373, Dar es Salaam, Tanzania; 5 Cairns Public Health Unit, P.O. Box 1103, Cairns, Queensland, 4870, Australia; 6 Liverpool School of Tropical Medicine, Pembroke Place, Liverpool, L3 5QA, UK

**Keywords:** *Anopheles gambiae*, *Anopheles arabiensis*, Tanzania, LLINs, Insecticide resistance

## Abstract

**Background:**

Long-lasting insecticide treated nets (LLINs) and indoor residual house spraying (IRS) are the main interventions for the control of malaria vectors in Zanzibar. The aim of the present study was to assess the susceptibility status of malaria vectors against the insecticides used for LLINs and IRS and to determine the durability and efficacy of LLINs on the island.

**Methods:**

Mosquitoes were sampled from Pemba and Unguja islands in 2010–2011 for use in WHO susceptibility tests. One hundred and fifty LLINs were collected from households on Unguja, their physical state was recorded and then tested for efficacy as well as total insecticide content.

**Results:**

Species identification revealed that over 90% of the *Anopheles gambiae* complex was *An. arabiensis* with a small number of *An. gambiae s.s.* and *An. merus* being present. Susceptibility tests showed that *An. arabiensis* on Pemba was resistant to the pyrethroids used for LLINs and IRS. Mosquitoes from Unguja Island, however, were fully susceptible to all pyrethroids tested. A physical examination of 150 LLINs showed that two thirds were damaged after only three years in use. All used nets had a significantly lower (p < 0.001) mean permethrin concentration of 791.6 mg/m^2^ compared with 944.2 mg/m^2^ for new ones. Their efficacy decreased significantly against both susceptible *An. gambiae s.s.* colony mosquitoes and wild-type mosquitoes from Pemba after just six washes (p < 0.001).

**Conclusion:**

The sustainability of the gains achieved in malaria control in Zanzibar is seriously threatened by the resistance of malaria vectors to pyrethroids and the short-lived efficacy of LLINs. This study has revealed that even in relatively well-resourced and logistically manageable places like Zanzibar, malaria elimination is going to be difficult to achieve with the current control measures.

## Background

Long-lasting insecticide treated nets (LLINs) and indoor residual house spraying (IRS) are the main interventions for the control of malaria vectors in Zanzibar. The Zanzibar Malaria Control Programme (ZMCP) started free LLIN distribution in 2006 targeting mainly pregnant mothers and children under the age of five years [1]. In 2008 every household in Zanzibar received two LLINs (Zanzibar Malaria control Programme, 2009 unpublished report) and, since 2006, six rounds of IRS have been conducted with lambda-cyhalothrin (ICON 10WP/CS) giving greater than 90% coverage of all dwellings (Zanzibar Malaria control Programme, 2011 unpublished report).

These two vector control interventions target indoor resting and indoor feeding mosquitoes [2]. The combination of IRS and LLINs with other interventions and planning tools (case management, intermittent preventive treatment, behavioral change communication, surveillance and monitoring) have resulted in a dramatic reduction of malaria prevalence in Zanzibar from 40% in 2005 to between 0.2 and 0.5% in 2011/2012 (Tanzanian Health Ministry Information System, 2012 unpublished report).

A major concern for many malaria vector control programmes in sub-Saharan Africa is that malaria mosquitoes are increasingly developing resistance to insecticides [3,4]. Pyrethroids are the most common insecticides for malaria vector control and the only insecticides used for LLINs, but their effectiveness is being threatened due to the emergence of resistance [5-10]. Monitoring the susceptibility of vectors to insecticides is therefore essential for predicting the sustainability and efficacy of these control tools [11].

The ZMCP therefore conducts routine insecticide efficacy monitoring to assess the susceptibility of malaria vectors on Pemba and Unguja, the two main islands of Zanzibar. In 2002, *Anopheles gambiae s.l*. from Unguja was fully susceptible to DDT, deltamethrin and lambda-cyhalothrin (Zanzibar Malaria control Programme, 2002 unpublished report). In 2008, *An. gambiae s.l*. from both Zanzibar islands were susceptible to deltamethrin, lambda-cyhalothrin and bendiocarb, however, there were indications of low levels of DDT resistance on Pemba (96.5% mortality) but not on Unguja (Zanzibar Malaria control Programme, 2008 unpublished report).

LLINs are one of the most powerful weapons in the fight against malaria. If properly used, these nets inflict a lethal (insecticidal) effect as well as forming a physical barrier against malaria transmitting mosquitoes [2,3]. Community-wide use of LLINs can affect vector density and longevity over large areas. *Anopheles* mosquitoes are forced to find alternative non-human hosts and ultimately this reduces mosquito survival and malaria transmission [12].

The aim of the present study was to assess the susceptibility status of malaria vectors against the pyrethroids used on LLINs and IRS and to determine the durability and efficacy of LLINs on the island. The World Health Organization (WHO) requires that LLINs remain effective after 20 standard washes and last for three years under field conditions [13]. However, washing practices, drying methods and climatic conditions can vary significantly from one place to another and all reduce the amount of active insecticide on the net. Consequently, aged nets may lose efficacy, particularly against pyrethroid-resistant mosquitoes, and may exacerbate selection for resistance, thus having serious implications for malaria transmission [10,14].

## Methods

### Study sites

**Figure 1:**
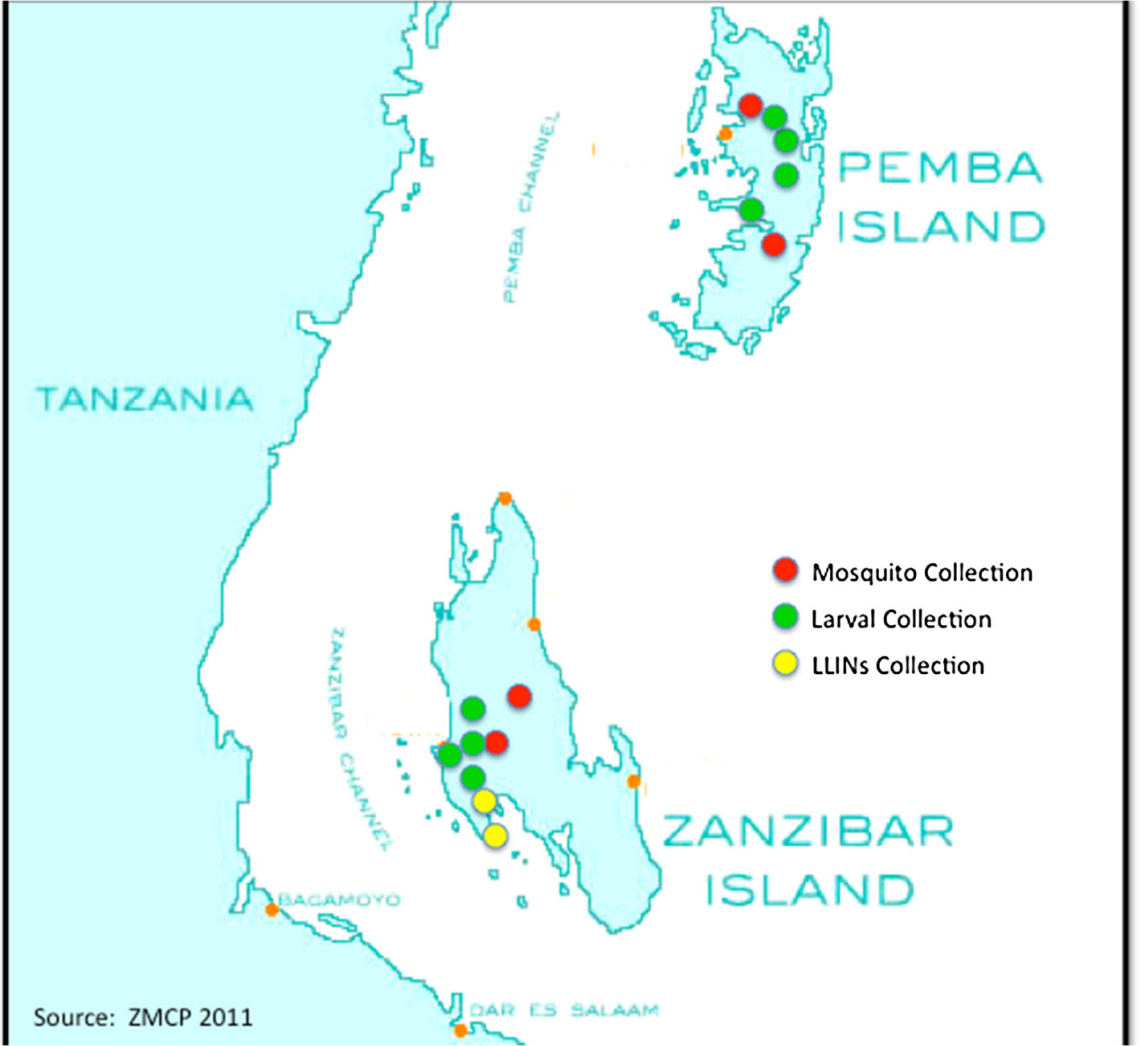
Map of the Zanzibar islands showing collection areas.

The study was conducted in the West district of Unguja that covers an area of 209 km^2^ with an estimated population of 262,676 people, and North Pemba which covers an area of 478 km^2^ with an estimated population of 221,386 people (Zanzibar 2002 Population and Housing census) (Figure 1).

### Mosquito sampling

Mosquito larvae were collected from various breeding sites. On Unguja, these sites were in rice-growing areas while on Pemba they were from sites around homesteads, rain pools, rice fields and waterlogged areas. The larvae were reared to adults in the insectary and pupae transferred to holding cages daily for adult emergence.

### Insecticide resistance assays

Four insecticides were tested on the islands of Unguja and Pemba (lambda-cyhalothrin 0.05%, permethrin 0.75%, deltamethrin 0.05% and bendiocarb 0.1%). On Pemba alone, an additional two insecticides were assessed (DDT 4% and malathion 5%). These were not tested on Unguja due to lack of sufficient mosquito samples. All tests were conducted on the above diagnostic dosages following WHO guidelines [15]. The first round of testing was completed in June 2010 on both islands and repeated in December 2010 and April 2011 for Pemba Island. A maximum of twenty-five, 2–5 day old, non-blood fed *Anopheles* females were inserted into exposure tubes and exposed to the insecticide impregnated papers for 60 min. At the end of the 60 min exposure, mosquitoes were transferred to holding tubes and provided with a 10% sugar solution. Mortality was noted after 24 hours. Control mosquitoes (25 per tube per insecticide) were handled in the same way as test mosquitoes but were not exposed to insecticides. WHO recommends that 100 mosquitoes be tested per insecticide [15] but this was not possible in December 2010 due to decreased mosquito populations on Pemba Island. Assays were conducted at 27 ± 2°C and 80 ± 10% relative humidity. Species identification using the polymerase chain reaction assay [16] was performed on selected samples at the Ifakara Health Institute, Tanzania.

### Long-lasting insecticide-treated nets

**Figure 2:**
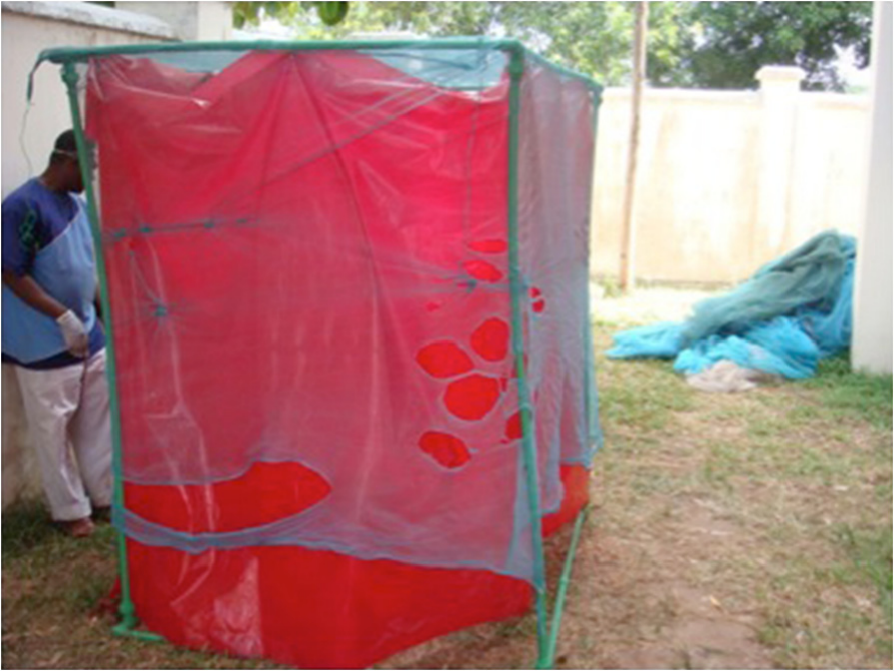
Damaged LLINs being examined on a plastic frame showing distribution of large holes (>5 cm) and knots as a method of net repair.

One hundred and fifty (150) LLINs were collected from randomly selected households in Kombeni and Nyamanzi villages in the West district of Unguja in May 2011. All the nets tested in this study were distributed by the ZMCP in 2008. The collected nets were replaced with new LLINs from ZMCP. Each collected net was packed in a clean plastic bag, labelled for identification and taken back to the ZMCP laboratory for further investigation. All LLINs were examined to identify the brand and manufacturer. The number of washes and the drying methods utilised were recorded using questionnaires. To facilitate physical evaluation the nets were draped over a cube-shaped frame (about the size of the net – 160x150x180cm) that was wrapped around with a red plastic sheet to facilitate a clear contrast with the net (Figure 2). The net was examined carefully and all tears and holes were measured. Seam failures were also recorded.

Evidence of repairs to the net fabric, type of repair and in the case of sewn repairs, length of stitching on repair was also recorded. Based on previously published criteria [14], nets were “undamaged” if they fulfilled the following criteria: less than 20 holes, of less than 2 cm in diameter; < 5 holes of 2–5 cm in diameter; and < 2 holes, > 5 cm in diameter.

A subset of 48 nets was randomly selected from the collection to test their insecticidal efficacy on mosquitoes. The selected nets had been washed six (n = 6), 12 (n = 17) and 36 (n = 25) times. These were tested in parallel with 10 new LLINs to demonstrate their comparative efficacies. Both were tested against susceptible *Anopheles gambiae s.s*. from the ZMCP insectary (originated from the National Institute for Medical Research (NIMR), Muheza, Tanzania) and wild caught *An. gambiae s.l.* from Pemba.

**Figure 3:**
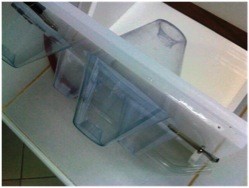
Double cone set up. Photograph courtesy of Dr P Guillet.

A double cone method for assessing mortality and knockdown was used to evaluate the LLIN efficacy. In this method 4 pairs of cones, each pair sandwiching an area of netting, were supported by two pieces of cardboard. The assemblage was held in place using clips (Figure 3). Mosquitoes were introduced into one cone and those that passed through the relatively large Olyset mesh (870 holes /100 cm^2^), were contained by the other. This technique reflects the behavior and insecticide exposure that occurs around an Olyset net more realistically than single cone designs in which the large mesh size might allow mosquitoes to rest without contacting the netting threads, or in which the mesh is doubled over and so decreases the mesh size but increases the dose per area. For every 10 replicates of five mosquitoes, four replicates of five mosquitoes were also run as control groups [13]. Untreated nets from local retailers were used for control groups. These nets are normally sold untreated and insecticide for their treatment supplied separately. A 30 cm^2^ piece of netting was cut from the long side of each net and tested using a susceptible *An. gambiae s.s.* colony from the ZMCP insectary and *An. gambiae s.l.* field mosquitoes from Pemba where resistance had been shown in earlier tests. Five female mosquitoes (2–5 days old non blood fed) were introduced into each cone for three minutes. Mosquitoes were then transferred to paper cups and provided with a 10% glucose solution [13]. The test was repeated 10 times for each of the 58 selected LLINs and mortality recorded after 24 hours. The cone tests were carried out at 27 ± 2°C and 80 ± 10% Relative Humidity [13].

Insecticide retention analysis on LLIN fibers was undertaken at the laboratories of the Center for Disease Control and Prevention Atlanta, Georgia, USA. Gas chromatography (GC) was used to determine the amount of insecticide on each LLIN specimen. The net samples were cut to 20x20 cm pieces and weighed. The specimens were then stored in individual 50 ml stoppered flasks. Five milliliters of triphenyl phosphate and 45 ml heptanes were added to each flask and the specimens homogenized. After shaking, 0.9 ml volume of this extract was added to the injection vial to determine the amount of permethrin retained within the LLIN fibers and on the surface of the nets (Protocol available at http://www.cipac.org/cipacpub.htm). A total of 60 LLINs (10 new and 50 field collected, the same net samples used in bio-efficacy testing) were analyzed using the GC method.

### Data analysis

Susceptibility of mosquitoes to insecticide was scored according to the WHO protocol [15]: 98-100% mortality indicates susceptibility, 80-97% mortality indicates the presence of resistance that needs to be confirmed and <80% mortality indicates resistance. Abbott’s formula is used to calculate corrected mortalities for all bio-efficacy tests where control mortality is between 5 and 20% [13,15]. Descriptive measurements for the physical state of the nets were collated. These described whether nets were new or old, the numbers and percentages holed, and the numbers of repairs undertaken. Non-parametric Mann–Whitney tests were used to compare permethrin concentrations in new versus washed nets. Kruskal-Wallis test was used to compare the impact of number of washes on mosquito mortality and permethrin concentration in nets. Bio-efficacy results of LLINs were analyzed based on the WHO protocol: minimum effectiveness is KD60 ≥75% or mortality ≥ 50% whereas optimal effectiveness should be KD60 ≥ 95% or mortality ≥ 80% [13].

## Results

### Species identification

Molecular identification of species tested in 2008 revealed that of the samples that amplified with PCR on Unguja (n = 300), 79% were *An. arabiensis*, 19% *An. merus* and 2% *An. gambiae s.s.* On Pemba (n = 155), where sampling was done from breeding sites that may have contained salt water, 38% were *An. merus* while *An. arabiensis* and *An. gambiae* were in equal proportions (31% each) (Zanzibar Malaria control Programme, 2008 unpublished report). However, of the samples that amplified with PCR in 2010, on Unguja (n = 83, June collections only) 90% were *An. arabiensis*, 5% *An. gambiae s.s.* and 5% *An. merus*, while on Pemba (n = 1110, June + December collections) samples were identified as *An. arabiensis* (97%) and *An. merus* (3%). In 2011, results showed the same trend on Pemba (n = 96) with 99% of the specimens being *An. arabiensis* and 1% *An. gambiae s.s.* (Jones, unpublished data). These results and further tests carried out as part of routine monitoring activities by ZMCP confirm that *An. gambiae s.s.* numbers have been greatly reduced on both islands and *An. arabiensis* is now the main malaria vector in Zanzibar.

### Susceptibility tests

**Figure 4:**
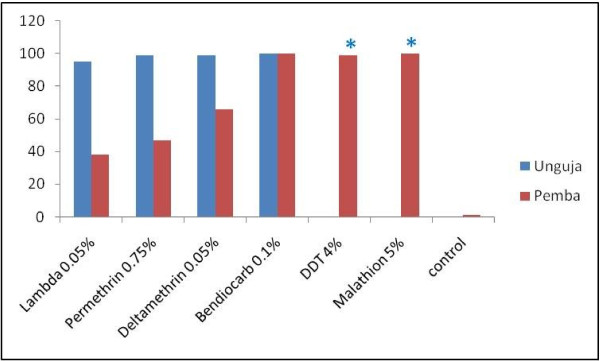
**Mosquito response (24 hr mortality) to different insecticides on Unguja and Pemba.** The results from Pemba are a summary of three testing periods. *DDT and Malathion were not tested on Unguja due to lack of sufficient samples.

Results from susceptibility tests showed strong resistance in *An. arabiensis* against all three pyrethroids tested on mosquitoes from Pemba (Table 1). In addition, 24 hr mortality on lambda-cyhalothrin decreased significantly (p = 0.006) over time from 48% in June 2010 to 46% in December 2010 and 9% in April 2011 (Table 2). Mortality at 24 hr also decreased significantly over time with deltamethrin (p = 0.002) while permethrin resistance remained stable (Table 2). On the island of Unguja however, mosquitoes were fully susceptible to all pyrethroids tested, although lambda-cyhalothrin showed some survival at 95% mortality (Table 2). Full susceptibility was recorded to bendiocarb on both islands and malathion and DDT on Pemba island (Figure 4).

**Table 1:** Species identification and insecticide susceptibility tests for mosquitoes collected on Pemba Island in December 2010

		***An. arabiensis***	***An. merus***	**Unidentified**	**Total**	**% 24 hr mortality**
Lambda-cyhalothrin 0.05%	Dead	26	5	3	74	46
	Alive	36	0	4		
Deltamethrin 0.05%	Dead	40	0	2	100	42
	Alive	48	7	3		
Permethrin 0.75%	Dead	28	16	7	89	57
	Alive	37	1	0		
Bendiocarb 0.1%	Dead	80	0	2	82	100
	Alive	0	0	0		
DDT 4%	Dead	95	0	3	98	100
	Alive	0	0	0		
Control	Dead	0	0	0	111	0
	Alive	101	5	5		

**Table 2:** **Percent mortality at 24 hrs (sample size) of *Anopheles gambiae s.ls *from Pemba and Unguja exposed to different classes of insecticides over different time periods**

	**Pemba**			**Unguja**
	June 2010	Dec 2010	April 2011	June 2010
Lambda-cyhalothrin 0.05%	48 (228)	46 (74)	9 (100)	95 (100)
Deltamethrin 0.05%	80 (247)	42 (100)	36 (100)	99 (100)
Permethrin 0.75%	46 (431)	57 (89)	51 (100)	99 (100)
Bendiocarb 0.1%	100 (259)	100 (82)	100 (100)	100 (100)
DDT 4%	-	100 (98)	100 (100)	-
Malathion 5%	-	-	100 (100)	-
Control	2 (325)	0 (111)	0 (150)	0 (100)

### Durability of nets

Of the 150 nets collected, 149 were Olyset™ Nets and the remaining one was a PermaNet in poor condition, whose version could not be identified. One Olyset net was found in its original package, i.e. the net had never been used since it was distributed three years previously. The mean number of washes of the 148 Olyset™ Nets was 23 (range 3–36). Nets were hung to dry either in shaded places or inside houses (60%), however, the remaining 40% of nets were dried in direct sunlight, against WHO recommendations [13]. A physical examination of the 148 used Olyset nets revealed a total of 1298 holes with a diameter >0.5 cm from 126 nets (average of 10.3 holes per holed net). More than 61% of these holes were located on the lower or bottom part of the nets and 4.5% were found on the LLINs roof. Up to 100 nets had holes larger than 5 cm (Figure 2) with an average of 5.3 holes/holed net and there were 41 seam failures identified from 18 LLINs. These results show that 68% of nets were considered damaged after only three years in use according to the published criteria [14].

### Net efficacy and insecticide content

**Figure 5:**
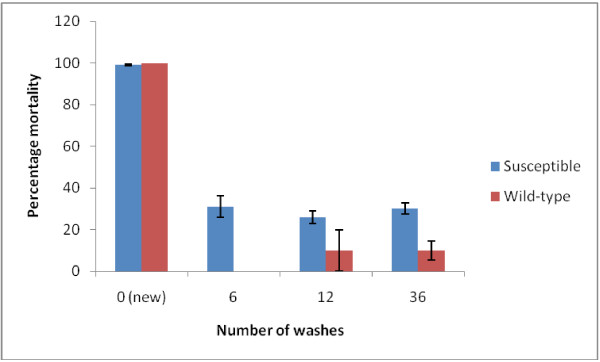
**Comparison of the number of LLIN washes on the mortality of susceptible (*An. gambiae s.s. *colony from the National Institute for Medical Research, Muheza) and wild mosquitoes (mostly *An. arabiensis *) from Pemba Island.** Unwashed = 10 new nets; 6 washes = 6 used nets; 12 washes = 17 used nets; 36 washes = 25 used nets.

Cone bioassays of the 58 nets selected for efficacy testing revealed that colony *An. gambiae s.s.* from the ZMCP insectary were fully susceptible to the new nets (100% mortality at 24 hrs). Wild-type mosquitoes from Pemba (almost exclusively *An. arabiensis*) were also susceptible to new nets (99.2% mortality at 24 hrs). However, the efficacy of used nets (washed 6–36 times) decreased significantly (p < 0.001) against both susceptible and wild-type mosquitoes but there was no significant difference between the number of washes and the mortality response (p > 0.05, Figure 5). There was a significantly higher proportion of mortality in susceptible than wild mosquitoes when using old/washed nets (p < 0.001, Figure 5). In all cases the nets did not reach the optimal effectiveness criteria with mortality well below 50% [13] after only three years in use.

Insecticide retention analysis showed the mean permethrin concentration of new LLINs to be 944.2 mg/m^2^_,_ whereas used LLINs had a significantly lower mean permethrin concentration of 791.6 mg/m^2^ (p < 0.001). As observed for cone assays, for washed nets, the number of washes did not have a significant impact on permethrin concentration (p > 0.05).

## Discussion

Zanzibar’s efforts in malaria vector control, largely based on IRS and LLINs, have contributed to a substantial decrease in malaria transmission over the years. These great achievements have raised hopes for the islands to enter malaria pre-elimination and elimination phases. However, results reported here raise serious concerns and demonstrate the substantial challenges that threaten the sustainability of the gains achieved.

The apparent near elimination of *An. gambiae s.s.* on the island has been observed in other parts of sub-Saharan Africa [17] and is usually attributed to the success of IRS and LLINs in controlling the endophilic and anthropophilic *An. gambiae s.s.* However, this leaves *An. arabiensis,* which is an equally efficient malaria vector, feeds indoors and outdoors, on human and non-human hosts and can rest outdoors [18], therefore is not optimally controlled by indoor interventions. The control of *An. arabiensis* for malaria elimination will require more tools in addition to LLINs and IRS.

In Zanzibar, IRS has been conducted for over six years now with more than 90% coverage of the island using lambda-cyhalothrin. LLINs impregnated with permethrin have been rolled out for more than three years to every household in Zanzibar. The decision to use the same class of insecticide (pyrethroid) for both IRS and LLINs at the same time has undoubtedly increased the selection pressure on the vectors to develop insecticide resistance [19].

Results from the standard WHO resistance assays in this study revealed a very pronounced and increasing pyrethroid resistance in *An. arabiensis* on Pemba Island over three collection periods within one year. Thirty years ago, *An. gambiae s.s.* was shown to be resistant to DDT on Pemba [20], while Lines and Nassor [21] reported the spread of DDT resistance to Unguja island. However, the current tests showed a 100% susceptibility to DDT for wild mosquitoes on Pemba, indicating that the lack of IRS using DDT, i.e. no DDT selection pressure, might have allowed the population to revert to the susceptible state. This suggests that the pyrethroid resistance now being documented is mediated by a metabolic and not a target site mechanism (cross-resistance between DDT and pyrethroids often suggests the presence of a *kdr* mutation [3,19]). It is unclear why pyrethroid resistance in Zanzibar is highly prevalent on Pemba while the mosquitoes on Unguja remain largely susceptible. The ZMCP implements the same vector control interventions on both islands and the distribution of malaria vector species is more or less the same (predominantly *An. arabiensis*).

Although the question of keeping livestock was not investigated in our study, farmers in many areas use pyrethroids for the control of ticks and other veterinary pests. This may contribute to the selection pressure for cattle feeding mosquitoes such as *An. arabiensis* and might accelerate the emergence of resistance. However, it is believed that there is no significant difference in keeping livestock between the two islands of Zanzibar (personal communication from the Zanzibar Veterinary Officer). In addition, heavy use of similar classes of insecticides in agriculture may contribute to the selection for insecticide resistant mosquitoes [22,23]. More investigations are required, therefore, to ascertain the origin and mechanism of resistance on Pemba and the difference between the two islands. Moreover, it is important to regularly assess the efficacy of lambda-cyhalothrin, which shows 5% survival in *An. arabiensis* on Unguja that may quickly become a serious resistance problem as it has on Pemba.

Physical inspection of LLINs distributed in early 2008 by ZMCP in the West district of Zanzibar has shown that 68% of the nets were in poor physical condition and below standard [14]. This could be attributed to the structure of the beds used in the villages, the common type being that of wood connected by metal clamps with protruding bolts from all four corners of the frame. Another type of bed that is locally made uses wood branches connected with rope. These kinds of beds damage the nets very quickly, particularly on the lower portion where the nets are touching the bed frame or are tucked under the mattress. Similar results were found in Ghana and rural Uganda where the number of holes increased towards the lower part of the nets [14,24]. Holes on the upper portion of the nets could be caused by tearing with sharp pointed sticks and other household materials. In some cases, burning was another cause of net damage as a result of exposure to naked flames from wood fires and kerosene lamps. Seam failure was responsible for damage found on the upper portion of a small number of the nets. The extent and size of holes and the frequency of seam failures demonstrates the need to raise the performance requirements for LLINs in terms of both fabric quality and seam strength [14].

In this study, two thirds of LLINs were damaged within three years of distribution. In the highlands of Ghana 84.8% of the nets were intact after 4–5 years of field use but only 56.6% of nets in lowland villages were intact 3–4 years after distribution [14]. In another study conducted in Lao, about 40% of nets were in poor physical condition after 2–3 years of field use [25]. These results suggest that net longevity is highly dependent on where they are being used and the type of bed structure.

While the present study showed (based on the cone bioassays) that new unwashed Olyset™ nets were completely effective against laboratory reared and wild-caught malaria vectors, the probability of LLINs being effective for vector control for any length of time is very low considering how rapidly the efficacy of the nets was reduced with washing (Figure 5). The mortalities achieved on the washed nets for the fully susceptible *An. gambiae s.s.* laboratory colony were far less than the minimum effectiveness recommended by WHO [13]. The use of wild-caught anophelines (largely *An. arabiensis*) showed even lower mortalities after only six washes indicating a serious lack of efficacy in the presence of pyrethroid resistant vectors. While there is some doubt about the reality of 25 nets being washed 36 times over 3 years, the results are no different to the groups that were washed only 6 or 12 times. Other studies using the same WHO cone method have revealed that LLINs had a knockdown rate of 95% and mortality rate of 80% after 36 months of field use in Uganda [24]. Similar results were observed after three years of net distribution in Buie and Fentalie districts of Ethiopia [26] and in a Tanzanian village after seven years of Olyset™ use [27]. The current results also contradict previous findings in Tanzania reporting that old Olyset nets that had been in domestic use for four years were as good as new nets [28,29]. However, WHO [30] reported in an overview of the variation of mortality and knockdown as a result of cone tests (on three year old nets collected from Angola, Ghana, Kenya, Madagascar, Togo and Zambia), that only 33% of 120 nets tested fulfilled the bioassays criteria. Furthermore, when the 80 nets that failed on the bioassay cone tests were subjected to a tunnel test, only 61% fulfilled the WHOPES criteria.

The insecticide retention analyses on Olyset™ nets carried out in the present study showed that they retained high permethrin concentrations within the fibers, but significantly less than new nets. The important aspect in mosquito mortality induced by LLINs is the surface concentration of insecticide, which is affected by a number of factors including frequent washing [31]. The present study revealed that 40% of nets were dried in direct sunlight, which might also have affected the performance of the nets. There was no significant difference in mortality on LLINs washed between six and thirty-six times but the reduction in efficacy between unwashed and those washed was highly significant. Although reports on washing frequency are not always accurate, the most significant and worrying finding of this investigation is that regardless of the number of washes, LLINs failed every criteria for efficacy within only three years of use.

Based on the low malaria prevalence in Zanzibar, the question has been raised as to when the country-wide indoor residual spraying, which is expensive, should be stopped. The decision would be based on both entomological and parasitological findings. If IRS is stopped, LLINs would be the only remaining strong vector control tool to combat the disease at this critical stage when Zanzibar is approaching the pre-elimination phase. The very low mortality achieved by old used nets tested on susceptible *An. gambiae* s.s and compounded even further by resistance in wild mosquitoes in Pemba, showed that the LLINs distributed in 2008 were no longer effective against malaria vectors in Zanzibar after three years of use. The necessity for net replacement therefore, should be considered well before three years, instead of the five years suggested by the manufacturer. More importantly, the evolution of pyrethroid resistance in the Zanzibar vector population calls for urgent implementation of insecticide resistance management using different classes of insecticides and currently this can only be achieved through IRS. The results reported here have prompted the ZMCP to start using bendiocarb for the current IRS campaigns. However, this switch is likely to be more costly for the program. Most of the cost of implementing IRS is operational (http://www.pmi.gov/resources/reports/irs_iqc08.pdf) and since bendiocarb has a relatively short residual life (2–3 months), this would require more rounds of annual spraying. The insecticide resistance mitigation plan for Zanzibar has, therefore, adopted a targeted approach to IRS with bendiocarb in areas with higher malaria prevalence instead of blanket coverage of the whole island. In parallel, it was also decided to redistribute new LLINs to every household in 2012. However, in order to remain effective, these LLINs would have to be replaced again before 2015.

## Conclusions

The sustainability of the achieved gains in malaria control in Zanzibar is seriously threatened by the resistance of malaria vectors to pyrethroids and the short-lived efficacy of LLINs. This study has shown that even in relatively well-resourced and logistically manageable places like Zanzibar, malaria elimination is going to be difficult to achieve with the current control measures.

## Competing interests

All authors declare that they have no competing interests.

## Authors’ contributions

KAH conceived the study and conducted this work as part of his Masters degree and participated in drafting the first manuscript. BOK participated in insecticide bio-assays testing on Pemba. SS supervised the analysis of insecticide retention on nets. ASA coordinated the work. GD and MC supervised the MSc work. SM and MC supervised the drafting of the manuscript. All authors read and approved the content of the manuscript.
